# Breakfast consumption was associated with suicidal ideation, plan, and attempt: a population-based cross-sectional study

**DOI:** 10.3389/fpubh.2024.1410499

**Published:** 2024-12-10

**Authors:** Yunshu Zhang, Keqing Li, Lili Zhang, Long Sun

**Affiliations:** ^1^School of Clinical Medicine, Hebei University, Baoding, Hebei, China; ^2^Hebei Provincial Mental Health Center, Baoding, Hebei, China; ^3^Centre for Health Management and Policy Research, School of Public Health, Cheeloo College of Medicine, Shandong University, Jinan, China; ^4^National Health Commission of China Key Lab for Health Economics and Policy Research (Shandong University), Jinan, China

**Keywords:** breakfast consumption, suicidal ideation, suicide plan, suicide attempt, population-based study

## Abstract

**Background:**

Although several studies have explored the association between breakfast consumption and suicidal behaviors among children and adolescents, such associations have been less frequently reported among adults and seniors.

**Method:**

This population-based cross-sectional study was conducted in Hebei Province, China. A total of 21, 376 community residents aged 18 years and older were interviewed. This study evaluated the breakfast frequency per week (BFF), full breakfast frequency (FBF), suicidal ideation, suicide plans, and suicide attempts of the participants. Sociodemographic variables, living alone, and sleep quality were also measured.

**Results:**

The prevalence of suicidal ideation, plans, and attempts were 1.4, 0.3, and 0.2%, respectively. Compared to ≥6 days/week BFF, 2–3 days/week BFF was positively associated with suicidal ideation (OR = 1.93, *p* < 0.01), suicide plan (OR = 2.73, *p* < 0.05), and suicide attempts (OR = 3.46, *p* < 0.05). In addition, participants with 2–3 days/week FBF was also at higher risk of suicidal ideation (OR = 2.06, *p* < 0.001), comparing with never FBF.

**Conclusion:**

The reported prevalence of suicidal ideation, plans, and attempts were lower compared to other countries. Lower breakfast frequency was positively associated with suicidal behaviors, and participants with 2–3 days/week of full breakfast consumption were also at a higher risk of suicidal ideation.

## Background

The World Health Organization (WHO) reports that more than 700,000 people die by suicide every year, and many more people attempt suicide for every suicide (1). In China, although suicide rates have decreased in recent years (2), suicide remains the main cause of death. The potential years of life lost due to suicide also ranked in the top 10 among all causes of death (3). Suicide is an important social and public health problem in China and other countries.

In recent years, many studies have explored the factors associated with suicidal behaviors (4–6), which have been shown to be related to a series of social, psychological, and behavioral factors (7–9), such as gender (10), childhood trauma (11), substance disorder (12). Recently, several studies have shown that breakfast consumption is associated with suicidal behaviors among adolescents. For example, a positive association between skipping breakfast and suicide attempts was observed by the Korean Youth Risk Behavior Survey (13–16). Skipping breakfast has also been shown to be positively associated with suicidal ideation, plans, and attempts in the National Youth Risk Behavior Survey of the United States (17–19). Although the associations between breakfast consumption and suicidal behaviors have been supported among adolescents, they have been less explored among adults and seniors worldwide.

In previous studies, the mechanism underlying the association between breakfast consumption and suicidal behaviors among children and adolescents has been explained by the following assumptions. The first and foremost explanation is emotional status (17). Previous studies have shown that skipping breakfast is positively associated with a series of emotional states such as depression and anxiety (20–22). Furthermore, considering the strong association between emotional status and suicidal behaviors (23, 24), skipping breakfast and suicidal behaviors could also be linked. Another explanation is brain metabolism. The demand for glucose in brain metabolism among children and adolescents is higher than that among adults and seniors. Skipping breakfast could cause a lack of glucose in children and adolescents and may further cause hormonal abnormalities (25–27), which are risk factors for suicidal behavior (28, 29). Although both explanations are reasonable, they may raise doubts about the relationship between skipping breakfast and suicidal behavior among adults and seniors.

To fill these gaps, a population-based cross-sectional study was conducted to explore the associations among community residents aged >18 years. The first was to explore the association between breakfast frequency and suicidal behaviors among community residents. The second aim was to explore the association between full breakfast frequency and suicidal behaviors among adults and seniors. If these associations could be established, the associations between breakfast frequency and suicidal behaviors can not only be generalized to the entire population but could also provide stronger evidence for public health policy interventions aimed at suicide prevention in China and other countries worldwide.

## Methods

### Study sample and design

A cross-sectional study design was employed to investigate a community-dwelling population between August and October 2018 in Hebei Province, China. Hebei is an industrial and agricultural province in northern China, with approximately 75 million inhabitants as of 2021 (30). A multistage stratified cluster sampling method was used to select the samples using the following steps. First, five cities were randomly selected from the 11 cities of Hebei Province. Second, among the five cities, three rural counties and one urban district were randomly selected. Third, in all the selected counties and districts, one township or subdistrict was randomly selected. Fourth, one village or neighborhood was randomly selected from each township or subdistrict. Finally, 15 villages (five cities × three counties × one township × one village) and five neighborhoods (five cities × one district × one sub-district × one neighborhood) were selected for this study. In the selected villages and neighborhoods, all community residents aged 18 years and older were invited to participate in the interviews. Finally, 21,376 valid questionnaires were analyzed, with a valid response rate of 73.25% (21,376/29,182). After the calculation of minimum sample size, this study needed 6,198 participants, with an increasing with 50%, the minimum sample size was 9,297, and the sample size in this study was enough for the analyses.

### Interview procedure

Data were collected via face-to-face interviews. One interviewer would interview one participant each time. Totally, there were 62 interviewers, who participated this study, and the interviewers were comprised of village doctors, undergraduate medical college students, and graduate students. All interviewers received training for one day to ensure thorough understanding of the research and questionnaire. Participants were scheduled for an interview after signing an informed consent form. On the night of the interviews, the interviewers were asked to check each other’s questionnaires. For each village or neighborhood, one supervisor was employed to further check the questionnaire. The interviewer was asked to revisit or call the interviewees to address missing data and logical errors. The Institutional Review Board (IRB) of the Sixth People Hospital of Hebei Province approved the study protocol prior to data collection. Written informed consent was obtained from all participants.

### Measures

#### Suicidal ideation, plan and attempt

Three questions were used to evaluate suicidal behavior. Suicidal ideation was evaluated based on whether the participants had ever seriously considered killing themselves. Participants with positive responses were asked whether they ever planned their suicidal behaviors and whether they ever attempted suicide. The answers to all three questions were yes or no. These three questions are widely used to evaluate suicidal behaviors worldwide, such as in the National Comorbidity Survey Replication (NCSR) (31) and many other studies (9, 23, 32).

#### Breakfast consumption

In this study, breakfast consumption considered two variables: breakfast frequency (BFF) and full breakfast frequency (FBFF). BFF was evaluated using the question, “What is the frequency for your breakfast?” The responses were 6 days/week, 4–5 days/week, 2–3 days/week, and 1 day/week. FBFF was also evaluated using the question “What is the frequency for your very full breakfast?” The answers were ≥ 4 days/week, 2–3 days/week, ≤ 1 day/week, and never.

#### Social-demographic variables

Both male and female participants were included in the study. Age was calculated using birth data. Ethnicity was categorized as Hans, Huis, and Manchus. Due to the low percentages of the last three responses, Ethnicity was recorded into Hans and others. Academic degree was evaluated as the highest academic degree received by the participants. The answers were illiterate, elementary school, middle school, high school, bachelor’s degree, and above. Because of the lower responses for the last two answers, academic degree was recorded as illiteracy, elementary, middle school, high school, and above. Married status was evaluated as single, married, or other, and the last answer contained divorced, widowed, remarried, or other. The region was analyzed in both rural and urban areas.

#### Living alone

Living alone was measured using a question about the number of people living with the participants (not including themselves). Participants who lived without others were classified as living alone, and those with other responses were classified as not living alone.

#### Sleep quality

The Chinese version of the Pittsburgh Sleep Quality Index (PSQI) was used to measure sleep quality (33). This scale is widely used to measure sleep quality in the world (34–36). This scale is comprised of seven dimensions. The sum of the scores in the seven dimensions was analyzed, with higher scores representing worse sleep quality.

### Statistical analysis

Data analyses were performed using SPSS for Windows 24.0 (web version). Means and standard deviations were described as continuous variables. Frequencies and percentages are described for categorical variables. The Student’s t-test or chi-square test was performed to compare the statistical significance of suicidal ideation, plans, or attempts. Logistic regression analysis was conducted to examine the association between related factors and suicidal behaviors. The independent variables with missing data were deleted, and the dependent variables with missing data were filling with the mean or modal number. All the statistical tests were two-tailed, and *p*-values <0.05 were considered as statistically significant.

## Results

In this study, data from 21,376 community residents were analyzed. In the study population, there were more females (11,537, 54.0%) and the mean age was 50.85 years. Detailed information on the samples can be found in the second row of Table 1. In this study, the prevalence rates of suicidal ideation, plans, and attempts were 1.4, 0.3, and 0.2%, respectively. Single analyses of the factors associated with suicidal behaviors were also conducted. Suicidal ideation was associated with gender (*χ*^2^ = 33.93, *p* < 0.001), age (*t* = 5.51, *p* < 0.001), academic degree (*χ*^2^ = 33.57, *p* < 0.001), married status (*χ*^2^ = 48.09, *p* < 0.001), living alone (*χ*^2^ = 31.84, *p* < 0.001) and sleep quality (*t* = 20.35, *p* < 0.001). Suicide plan was associated with age (*t* = 2.20, *p* < 0.05), married status (*χ*^2^ = 6.35, *p* < 0.05) and sleep quality (*t* = 7.30, *p* < 0.001). Suicide attempt was associated with sleep quality (*χ*^2^ = 3.58, *p* < 0.001). Detailed information is provided in Table 1.

**Table 1:** Sample description and single analyses for the factors associated with suicidal behaviors [Mean ± SD/n (%)].

Variables	Mean ± SD/ n (%)	Suicidal ideation		t/*χ*^2^	Suicide plan		t/*χ*^2^	Suicide attempt		t/*χ*^2^
		Yes	No		Yes	No		Yes	No	
N	21,376 (100.0)	289 (1.4)	21,087 (98.6%)	–	67 (0.3)	21,309 (99.7)	–	40 (0.2)	21,336 (99.8)	–
Gender				33.93***			1.41			0.02
Male	9,839 (46.0)	84 (0.9)	9,755 (99.1)		26 (0.3)	9,813 (99.7)		18 (0.2)	9,821 (99.8)	
Female	11,537 (54.0)	205 (1.8)	11,332 (98.2)		41 (0.4)	11,496 (99.6)		22 (0.2)	11,515 (99.8)	
Age	50.85 ± 16.30	56.10 ± 14.50	50.78 ± 16.31	5.51***	55.22 ± 14.48	50.84 ± 16.30	2.20*	48.98 ± 16.97	50.85 ± 16.30	−0.73
Ethnicity				0.34			2.36			1.14
Hans	20,094 (94.0)	274 (1.4)	19,820 (98.6)		60 (0.3)	20,034 (99.7)		36 (0.2)	20,058 (99.8)	
Others	1,282 (6.0)	15 (1.2)	1,267 (98.8)		7 (0.5)	1,275 (99.5)		4 (0.3)	1,278 (99.7)	
Academic degree				33.57***			7.10			2.57
Illiteracy	2,691 (12.6)	55 (2.0)	2,636 (98.0)		9 (0.3)	2,682 (99.7)		2 (0.1)	2,689 (99.9)	
Elementary	5,264 (24.6)	96 (1.8)	5,168 (98.2)		25 (0.5)	5,239 (99.5)		11 (0.2)	5,253 (99.8)	
Middle school	8,274 (38.7)	99 (1.2)	8,175 (98.8)		23 (0.3)	8,251 (99.7)		15 (0.2)	8,259 (99.8)	
High school or above	5,147 (24.1)	39 (1.8)	5,108 (99.2)		10 (0.2)	5,137 (99.8)		12 (0.2)	5,135 (99.8)	
Married Status				48.09***			6.35*			2.83
Single	1,548 (7.2)	13 (0.8)	1,535 (99.2)		3 (0.2)	1,545 (99.8)		2 (0.1)	1,546 (99.9)	
Married	18,487 (86.5)	230 (1.2)	18,257 (98.8)		55 (0.3)	18,432 (99.7)		33 (0.2)	18,454 (99.8)	
Others	1,341 (6.3)	46 (3.4)	1,295 (96.6)		9 (0.7)	1,332 (99.3)		5 (0.4)	1,336 (99.6)	
Region				1.55			1.31			2.85
Urban	5,100 (23.9)	60 (1.2)	5,040 (98.8)		12 (0.2)	5,088 (99.8)		5 (0.1)	5,095 (99.9)	
Rural	16,276 (76.1)	229 (1.4)	16,047 (98.6)		55 (0.3)	16,221 (99.7)		35 (0.2)	16,241 (99.8)	
Living alone				31.84***			1.45			0.28
Yes	1,193 (5.6)	38 (3.2)	1,155 (96.8)		6 (0.5)	1,187 (99.5)		3 (0.3)	1,190 (99.7)	
No	20,183 (94.4)	251 (1.2)	19,932 (98.8)		61 (0.3)	20,122 (99.7)		37 (0.2)	20,146 (99.8)	
Sleep quality	4.60 ± 3.54	8.76 ± 4.80	4.54 ± 3.48	20.35***	7.75 ± 4.96	4.59 ± 3.53	7.30***	6.75 ± 5.22	4.60 ± 3.53	3.85***

SD denotes to standard deviation. *p < 0.05; **p < 0.01; ***p < 0.001.

The results showed that 85.6% of the participants had breakfast for more than 6 days/week. Single analyses of the association between breakfast consumption and suicidal behaviors are presented in Table 2. In this study, breakfast frequency (BFF) and full breakfast frequency (FBFF) were analyzed. The results showed that BFF was associated with suicidal ideation (*χ*^2^ = 11.16, *p* < 0.05), plan (*χ*^2^ = 11.10, *p* < 0.05), and attempt (*χ*^2^ = 12.22, *p* < 0.01), respectively. FBFF was associated with suicidal ideation (*χ*^2^ = 15.01, *p* < 0.01).

**Table 2:** Single analyses for the associations between breakfast consumption and suicidal behaviors (*n* = 21,376).

Variables	n (%)	Suicidal ideation, n (%)		*χ* ^2^	Suicide plan, n (%)		*χ* ^2^	Suicide attempt, n (%)		*χ* ^2^
		Yes	No		Yes	No		Yes	No	
BFF				11.16*			11.10*			12.22**
≥6 days/week	18,307 (85.6)	238 (1.3)	18,069 (98.7)		48 (0.3)	18,259 (99.7)		28 (0.2)	18,279 (99.8)	
4–5 days/week	1,045 (4.9)	12 (1.1)	1,033 (98.9)		6 (0.6)	1,039 (99.4)		2 (0.2)	1,043 (99.8)	
2–3 days/week	832 (3.9)	22 (2.6)	810 (97.4)		6 (0.7)	826 (99.3)		5 (0.6)	827 (99.4)	
≤1 day/week	1,192 (5.6)	17 (1.4)	1,175 (98.6)		7 (0.6)	1,185 (99.4)		5 (0.4)	1,187 (99.6)	
FBFF				15.01**			1.05			1.92
≥4 days/week	5,678 (26.6)	68 (1.2)	5,610 (98.8)		15 (0.3)	5,663 (99.7)		13 (0.2)	5,665 (99.8)	
2–3 days/week	1953 (9.1)	45 (2.3)	1908 (97.7)		8 (0.4)	1945 (99.6)		5 (0.3)	1948 (99.7)	
≤1 day/week	3,546 (16.6)	48 (1.4)	3,498 (98.6)		11 (0.3)	3,535 (99.7)		7 (0.2)	3,539 (99.8)	
Never	10,199 (47.7)	128 (1.3)	10,071 (98.7)		33 (0.3)	10,166 (99.7)		15 (0.1)	10,184 (99.9)	

BFF denotes to breakfast frequency. FBFF denotes to full breakfast frequency. SD denotes to standard deviation. *p < 0.05; **p < 0.01; ***p < 0.001.

Logistic regressions were conducted to analyze the association between breakfast consumption and suicidal behaviors. The detailed results are presented in Table 3. For suicidal ideation, the associated factors were male sex (OR = 0.68, *p* < 0.01), elementary degree (OR = 1.60, *p* < 0.05), married status (OR = 0.64, *p* < 0.05), sleep quality (OR = 1.25, *p* < 0.001), 2–3 days/week BFF (OR = 1.93, *p* < 0.01), and 2–3 days/week FBF (OR = 2.06, *p* < 0.001). Suicide plan was associated with sleep quality (OR = 1.18, *p* < 0.001), 2–3 days/week BFF (OR = 2.73, *p* < 0.05), and ≤ 1 day/week BFF (OR = 2.34, *p* < 0.05). Suicide attempt was associated with illiteracy (OR = 0.16, *p* < 0.05), sleep quality (OR = 1.17, *p* < 0.001), 2–3 days/week BFF (OR = 3.46, *p* < 0.05), and ≤ 1 day/week BFF (OR = 2.89, *p* < 0.05).

**Table 3:** Logistical regressions for the associations between breakfast consumption and suicidal behaviors (*n* = 21,376).

Variables	Suicidal ideation	Suicide plan	Suicide attempt
Male	0.68 (0.52, 0.88)**	0.94 (0.56, 1.57)	1.05 (0.55, 2.00)
Age	1.00 (0.99, 1.01)	1.01 (0.99, 1.03)	0.96 (0.96, 1.01)
Hans ethnicity	1.29 (0.76, 2.21)	0.56 (0.25, 1.24)	0.49 (0.17, 1.41)
Academic degree (Ref. = High school or above)			
Illiteracy	1.24 (0.76, 2.02)	0.81 (0.29, 2.27)	0.16 (0.03, 0.81)*
Elementary	1.60 (1.05, 2.44)*	1.52 (0.67, 3.47)	0.53 (0.21, 1.32)
Middle school	1.40 (0.94, 2.06)	1.17 (0.54, 2.53)	0.54 (0.25, 1.20)
Married status (Ref. = Others)			
Single	0.72 (0.35, 1.47)	0.54 (0.12, 2.38)	0.18 (0.03, 1.17)
Married	0.64 (0.42, 0.99)*	0.59 (0.24, 1.43)	0.41 (0.13, 1.30)
Rural region	1.02 (0.74, 1.39)	1.26 (0.64, 2.47)	2.60 (0.97, 6.96)
Living alone	1.34 (0.84, 2.14)	0.90 (0.32, 2.58)	1.11 (0.26, 4.69)
Sleep quality	1.25 (1.22, 1.29)***	1.18 (1.12, 1.25)***	1.17 (1.08, 1.26)***
BFF (Ref. = ≥6 days/week)			
4–5 days/week	0.91 (0.50, 1.65)	2.34 (0.99, 5.55)	1.21 (0.29, 5.15)
2–3 days/week	1.93 (1.21, 3.07)**	2.73 (1.14, 6.56)*	3.46 (1.29, 9.27)*
≤1 day/week	1.12 (0.67, 1.88)	2.34 (1.02, 5.36)*	2.89 (1.06, 7.88)*
FBF (Ref. = Never)			
≥4 days/week	1.14 (0.84, 1.55)	1.03 (0.55, 1.92)	1.96 (0.91, 4.23)
2–3 days/week	2.06 (1.44, 2.93)***	1.41 (0.64, 3.09)	2.02 (0.72, 5.69)
≤1 day/week	1.19 (0.85, 1.68)	1.05 (0.52, 2.09)	1.57 (0.63, 3.92)
Constant	0.003***	0.001***	0.003***
*R* ^2^	0.13	0.07	0.07

BFF denotes to breakfast frequency. FBFF denotes to full breakfast frequency. SD denotes to standard deviation. *p < 0.05; **p < 0.01; ***p < 0.001.

## Discussion

This study has several findings. The reported prevalence rates of suicidal ideation, suicide plans, and suicide attempts were 1.4, 0.3, and 0.2%, respectively. The main aim of this study was to explore the association between breakfast consumption and suicidal behavior. The results indicated that a lower BFF was positively associated with suicidal ideation, plans, and attempts. In addition, participants with 2–3 days/week FBF were also in higher risk of suicidal ideation, comparing with never FBF.

In this study, the prevalence of suicidal behaviors was slightly lower than those reported in other studies. A meta-analysis reported that the prevalence of suicidal ideation and attempts in Ethiopia ranges from 1 to 55% and 0.6 to14%, respectively (37). Studies in Norway reported that the prevalence of suicidal ideation is approximately 15% (38). In China, a meta-analysis reported that the prevalence of suicidal ideation and attempts was 3.9 and 0.8%, respectively (39). Another study conducted in a metropolitan area reported that the lifetime prevalence rates of suicidal ideation, plans, and attempts were 3.1, 0.9, and 1.0%, respectively (40). All these studies remind us that the international prevalence of suicidal behaviors was much higher than in our studies, and the prevalence of suicidal behaviors in this study was slightly lower than in other studies conducted in China. This may be explained by the recent downward trend in suicide rates in China, particularly in rural regions (2).

The main aim of this study was to test the association between BFF and suicidal behaviors. The results supported that a lower BFF was positively related to suicidal ideation, planning, and attempts. These associations have been shown in children and adolescents in several studies (41, 42). In this study, the association between BFF and suicidal behaviors was extended to adults and seniors. For children and adolescents, one of the main explanations is the higher demand for glucose in the brain metabolism. Another explanation is the effect of mood problems. Lower BFF has been associated with mood problems (43), which is a risk factor for suicidal behaviors worldwide (23, 44). The supported association may also be explained by healthy lifestyle, which is a protective factor for suicidal behaviors (41, 45).

This study also found that FBF was associated with suicidal ideation, but not with suicidal plans and attempts. Bulimia nervosa is characterized by consumption of large amounts of food. Individuals with FRF may be at a higher risk of bulimia nervosa, which is an important risk factor for suicidal behavior (46, 47). However, FBF was not associated with suicidal plans or attempts. This may be due to the small sample size and number of patients in this study. Larger samples should be analyzed to explore the associations between suicide plans, attempts, and FBF in the future.

In this study, poor sleep quality was positively associated with suicidal behavior. The effects of sleep quality on suicidal behavior have been identified in many previous studies (44, 48, 49). Other risk factors for suicidal ideation were female sex and a lower academic degree. In previous studies, both gender and education level also have been identified to be associated with suicidal ideation in China (50, 51). China is one of the few countries with high female suicide rates worldwide (52, 53). The causal relationship between education and suicidal behavior has also been supported by previous studies (54).

This study had some limitations that should be considered when interpreting its findings. First, because of its cross-sectional design, causal relationships between breakfast consumption and suicidal behaviors could not be inferred. Second, the variables analyzed in this study were self-reported, and recall bias cannot be avoided. Finally, the factors associated with suicidal behaviors are complex. Although several related factors were controlled for to obtain these findings, many other related factors were not controlled in this study.

## Conclusion

Although there were some limitations to this study, to our knowledge, this is the first study to report an association between breakfast consumption and suicidal behaviors among adults and older adults. This study has several critical findings. First, the reported prevalence of suicidal behaviors was lower than those reported in previous studies. In addition, fewer BFF were positively associated with suicidal behaviors, and participants with 2–3 days/week of FBF were also at a higher risk of suicidal ideation. These findings extend our knowledge regarding the association between breakfast consumption and suicidal behaviors in adults and seniors. Considering breakfast consumption is a kind of healthy lifestyle, the findings in this study further imply the importance of healthy lifestyle on suicide prevention. Future studies can be conducted to explore the mechanism for the associations between breakfast consumption and suicidal behaviors.

## Data Availability

The raw data supporting the conclusions of this article will be made available by the authors, without undue reservation.
